# Does corneal epithelial thickness show the severity of psoriasis? SD-OCT study^☆^

**DOI:** 10.1016/j.abd.2022.11.006

**Published:** 2023-06-22

**Authors:** İrfan Botan Güneş, Berna Aksoy, Hakan Öztürk, Fuat Yavrum, Bediz Özen

**Affiliations:** a Department of Ophthalmology, Kocaeli Health and Technology University, Medical Park Kocaeli Hospital, Kocaeli, Turkey; b Department of Dermatology, Medical Park Kocaeli Hospital, Kocaeli, Turkey; c Department of Ophthalmology, University of Health Sciences, Tepecik Hospital, Izmir, Turkey; d Department of Ophthalmology, Alaaddin Keykubat University, Alanya, Turkey

**Keywords:** Epithelium, corneal, Psoriasis, Tomography, optical coherence

## Abstract

**Background:**

Previous studies have generally focused on dry eye test abnormalities and ocular involvements such as uveitis, and blepharitis in psoriasis. Psoriasis area severity ındex (PASI), which is used to assess psoriasis severity, is a time-consuming and complex tool.

**Objective:**

To evaluate the relationship between disease severity and central corneal epithelial thickness (CCET) in psoriasis.

**Methods:**

175 eyes of 175 psoriasis patients and 57 eyes of 57 healthy individuals as a control group was included in this study. Psoriasis patients were divided into three subgroups according to PASI score as < 10 mild, 10‒20 moderate and > 20 severe. CCET was measured by spectral domain-optical coherence tomography (SD-OCT), and mean values were recorded. Mean CCET values were compared between the psoriasis groups and the control group. Additionally, the relationship between PASI score and CCET was examined.

**Results:**

The mean CCET value was 58.06 ± 3.1 μm in the mild group, 60.10 ± 5.0 μm in the moderate group, 65.75 ± 6.3 μm in the severe group and 56.16 ± 3.1 μm in the control group. It was determined that the mean CCET value was significantly higher in all psoriasis groups compared to the control group (p < 0.001). The mean CCET value was significantly higher in the moderate psoriasis group than in the mild psoriasis group (p = 0.018), and in the severe psoriasis group compared to the moderate psoriasis group (p < 0.001). There was a strong positive correlation between PASI score and CCET (p < 0.001, *r* = 0.519).

**Study limitations:**

Cross-sectional design and a relatively small number of participants.

**Conclusions:**

There is a strong positive correlation between psoriasis severity and CCET. Contactless measurement of CCET by SD-OCT can be an indicator of psoriasis severity.

## Introduction

Psoriasis is a chronic inflammatory skin disease with a strong genetic predisposition and autoimmune pathogenic traits characterized by epidermal hyperproliferation and a relatively high epithelial basal turnover rate.^1^

Psoriasis typically affects the skin, but may also affect the joints and has been associated with a number of diseases. Inflammation is not limited to the psoriatic skin and has been shown to affect different organ systems. When compared to control subjects, psoriasis patients exhibit increased hyperlipidemia, hypertension, coronary artery disease, type 2 diabetes, and increased body mass index.^2^

In addition to these systemic diseases, ocular involvement is common, affecting 12% of cases.^3^, ^4^ Decreased corrected visual acuities, pterygium, cataract, blepharitis, keratitis, conjunctival hyperemia, and dry eye have been reported as common in patients with psoriasis.^5^, ^6^ In addition, punctate keratitis and corneal melting have been reported in psoriasis.^7^, ^8^ In inflammatory processes affecting the ocular surface, the cornea, and conjunctival epithelium are also potentially affected.^9^

The corneal epithelium is a non-keratinized squamous epithelium playing a very important role in protecting the eye and maintaining high optical quality, as it is the outermost layer.^9^, ^10^, ^11^ It has been found that the epithelium contributes 0.85 D alone in corneal refraction at the 3.6 mm diameter zone.^12^

Spectral-Domain Optical Coherence Tomography (SD-OCT) is a contactless technique that enables the measurement of corneal epithelial thickness and an accurate assessment of the corneal layers with great reliability and repeatability.^13^, ^14^, ^15^ SD-OCT is widely used in ophthalmology clinics today.

Psoriasis Area Severity Index (PASI) score is an objective method that scores the severity of psoriasis disease. In determining the PASI score, the affected body surface area, erythema, induration, and scaling are evaluated.^16^ On the other hand, PASI is a time-consuming and complex tool.^17^

The authors know that the literature contains no studies examining the relationship between the severity of psoriasis and corneal epithelial thickness. In this study, the authors investigated the thickness of the corneal epithelial SD-OCT thickness map in psoriasis patients according to PASI score compared to normal subjects.

## Material and methods

This cross-sectional case-control study was performed with the approval of the University of Istinye’s Medical Research Ethical Committee (approval number: 2/2021.K-67) according to the ethical principles of the Declaration of Helsinki. Written consent forms were received from all participants.

175 eyes of 175 patients with psoriasis and 57 eyes of 57 healthy participants (the Control Group [CG]) were included in this study. Only the right eyes of the participants were included in the study. Each participant underwent a complete ocular examination, including Schirmer’s test.^18^ Demographic data and anterior and posterior segment examinations were recorded in all cases.

The same specialist (Aksoy B) calculated and recorded the PASI score for each patient. Psoriasis patients were divided into three subgroups according to the PASI score: mild subgroup (PASI <10), moderate subgroup (PASI between 10 and 20), and severe subgroup (PASI >20).^19^, ^20^ The study excluded individuals with previous histories of ocular trauma or surgery, individuals having dry eye, corneal and conjunctival pathologies or active ocular diseases, individuals previously using long-term topical eye medications, and individuals using contact lenses.

**Figure 1:**
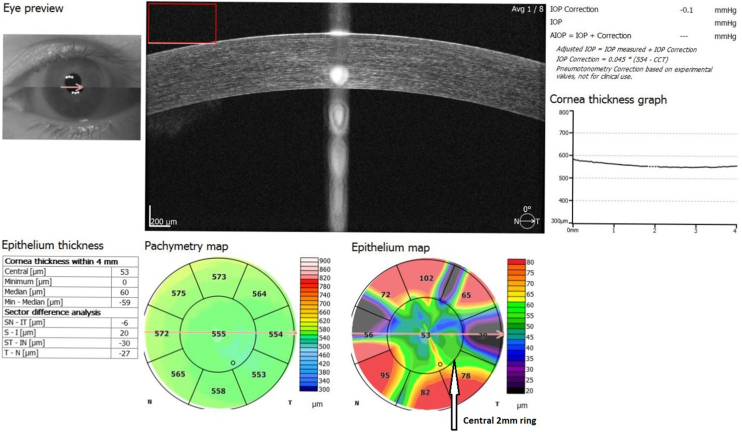
Mean corneal epithelial thickness maping with SD-OCT. Central 2-mm zone ring indicated by the white arrow. Mean corneal epithelial thickness in central 2-mm zone used in the analysis of measurements. Unit: μm

Measurements of the Corneal Epithelial Thickness (CET) were done by the same SD-OCT device (Optopol Technology sp.zo. Zawiercie Polska). OCT measurements were performed before the ophthalmological examination to avoid potential artifacts. The corneal epithelial thickness map of the patients was obtained using the cornea imaging mode of the SD-OCT device (Fig. 1). Thickness values of the corneal epithelium were examined within a circular area of diameter 4 mm. Corneal epithelial thickness value of the central area of 2 mm diameter was defined as the Central Corneal Epithelial Thickness (CCET).^21^

The same specialist (Gunes IB) performed all measurements. Measurements were done between 09.00 and 11.00 to avoid the effects of diurnal variation. Mean CCET values of mild, moderate, and severe psoriasis groups were compared with those of the control group. The psoriasis subgroup values were also compared.

The Statistical Package for Social Sciences version 20.0 was used in the statistical analysis of all data. The “Kolmogrov Simirnov” and “Shapiro-Wilk” tests examined the compatibility of numerical data and normal distribution. The “Mann-Whitney *U*” test was used for two independent groups and “Kruskal-Wallis H” test was used for more than two independent groups that did not show normal distribution. To detect significant differences between more than two independent groups, one of the post-hoc analyses, the Mann-Whitney *U* test with Bonferroni correction, was used. The Chi-Square test was used in the analysis of categorical variables among themselves. “Spearman Coefficient” was used in correlation tests. The results were analyzed at a 95% Confidence Interval and a p-value <0.05 was considered statistically significant.

## Results

The present study included 232 eyes of 232 participants. The mean age of the participants was 43.60 (min: 17, max: 88), of which 57.8 % were female (n = 134) and 42.2% were male (n = 98). The patients were divided into 3 groups according to the PASI score. The mild group included 64 eyes (27.6%), the moderate group included 58 eyes (25.0%), and the severe group included 53 eyes (22.8%). The control group included 57 eyes (24.6%). There was no significant difference in age and gender distribution between the groups in the study (p = 0.103, p = 0.242, respectively). Table 1 shows the demographic data of the patient groups and the control group.

**Table 1:** Demographic data, CCET values and PASI score of the groups

	Mild - PASI<10 (1)	Moderate - PASI between 10 and 20 (2)	Severe - PASI >20 (3)	Control (4)	p
	n = 64	n = 58	n = 53	n = 57	
Gender (F/M)	40/24	28/30	32/21	34/23	p = 0.242
Mean Age ± SD (range) years	39.69 ± 11.1	45.79 ± 12.6	45.15 ± 14.6	39.38 ± 11.4	p = 0.103
	(17‒66)	(27‒72)	(26‒88)	(17‒75)	
PASI score ± SD (range)	3.85 ± 0.26	13.69 ± 0.29	27.27 ± 1.04		p < 0.001
	(1.20‒9.00)	(10.80‒19.60)	(20.40‒49.20)		
CCET±SD μm (range)	58.06 ± 3.1	60.10 ± 5.0	65.75 ± 6.3	56.16 ± 3.1	1‒2: p = 0.018^a^
	(52‒66)	(52‒75)	(54‒80)	(51‒66)	1‒3: p < 0.001^a^
					1‒4: p = 0.001^a^
					2‒3: p < 0.001^a^
					2‒4: p < 0.001^a^
					3‒4: p < 0.001^a^

SD; Standard Deviation, n: Number of cases, F/M: Females/Males, PASI: Psoriasis Area Severity Index, CCET: Central Corneal Epithelial Thickness (μm).

a Two-way comparisons.

The mean corneal epithelial thickness was 58.06 ± 3.1 μm in the mild group, 60.10 ± 5.0 μm in the moderate group, 65.38 ± 5.8 μm in the severe group and 56.16 ± 3.1 μm in the control group.

**Figure 2:**
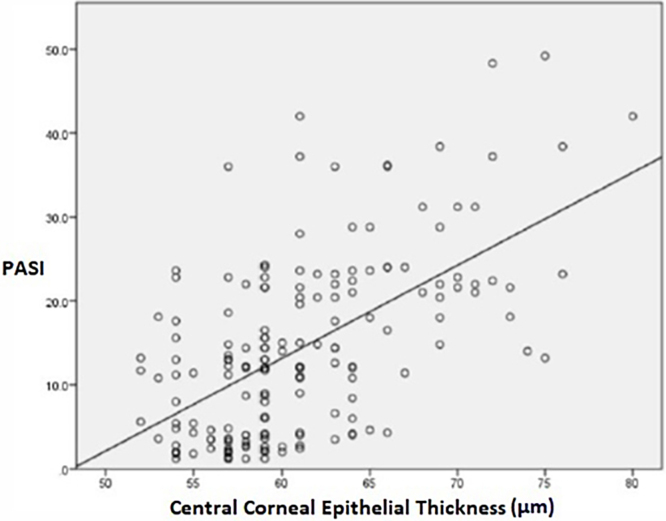
Corelation between PASI and CCET

The mean CCET values showed a statistically significant difference between the groups (p < 0.001). The mean corneal epithelium thickness in the control group was found to be significantly lower than all psoriasis groups (p < 0.001). In post hoc analysis performed among psoriasis groups, the mean CCET value was found to be significantly lower in the mild group compared to the moderate group (p = 0.018), and the mean CCET value was found to be significantly lower in the mild and moderate groups compared to the severe group (p < 0.001). In addition, there was a strong positive correlation between the PASI score indicating disease severity and corneal epithelial thickness (p < 0.001, *r* = 0.519) (Fig. 2).

## Discussion

The present study aimed to evaluate the thickness of the corneal epithelium in psoriasis patients using SD-OCT. Previous studies have generally focused on dry eye test abnormalities and ocular involvements such as uveitis, and blepharitis in psoriasis.^4^, ^5^, ^6^, ^7^ Unlike previous studies, this study evaluates the relationship between corneal epithelial thickness and disease severity in psoriasis patients.

Various studies have shown that the measurement of CCET with SD-OCT, which measures contactless with the eye, is reliable and reproducible.^15^, ^22^, ^23^ The mean CCET was examined in previous studies, with the application of impression cytology, in vivo confocal microscopy, or ultrasound.^24^, ^25^, ^26^ Various studies have widely used SD-OCT, benefitting from its lack of contact, accuracy, stability, and repeatability of results.^27^, ^28^

Ostadian et al. found the average CCET value of the normal population to be 56.64 ± 2.82 μm in measurements made with SD-OCT.^29^ Similar to this study, the mean corneal epithelial thickness was 56.16 ± 3.1 μm in this study’s control group.

Kanellopoulos et al.^30^ reported a positive correlation between corneal epithelial thickness and age. Samy et al. reported that CCET value decreases with age but not according to gender.^31^ The authors found no significant difference in mean corneal epithelial thickness according to gender and age in the patient and control groups.

Previous studies have described the involvement of the cornea in psoriasis as superficial punctate keratitis, opacities, recurrent erosions, ulcers, and scars.^5^, ^6^, ^7^, ^8^ In addition, Her et al.^6^ showed that dry eye is common in patients with psoriasis and that the tear film in these patients is relatively unstable because of ocular surface damage. The etiopathogenic mechanism of psoriasis affecting the ocular surface has not yet been clarified. In this study, corneal epithelial thickening was observed in psoriasis patients without eye involvement with psoriatic plaques. Eye involvement in psoriasis without psoriatic plaque may be a result of the psoriatic phenotype, which can induce immune-mediated inflammatory processes in organs other than the skin.^32^

PASI scoring is an objective method that shows the severity of psoriasis. When determining the PASI score, the affected body surface area, erythema, induration, and scaling are evaluated.^16^

The present study divided its psoriasis patients into three groups according to mild, moderate, and severe PASI scores. When the authors compared the mean corneal epithelial thickness of the patient groups and the control group, we found that the corneal epithelial thickness was significantly higher in the mild, moderate and severe patient groups compared to the control group. When the patient groups were evaluated among themselves, the average corneal epithelial thickness of the moderate and severe groups was found to be significantly higher than the mild group. In addition, a strong positive correlation was observed between disease severity and corneal epithelial thickness (p < 0.001, *r* = 0.519).

A study about the trypsin-like serine protease marapsin, emphasizes that marapsin expression is only found in the nonkeratinizing type of squamous epithelium in humans, such as the esophagus, tonsil, larynx, cervix, and cornea.^33^ These marapsin-expressing epithelia are all relatively thick, have a relatively high turnover rate, and are nonkeratinizing. In addition marapsin is not expressed in the keratinizing epithelium but is strongly induced when the epidermis undergoes hyperplasia and hyperproliferation, such as during wound re-epithelialization and in psoriasis. Marapsin expression is associated with squamous differentiation of keratinocytes, either constitutively in nonkeratinizing epithelia or under conditions of hyperproliferation and hyperplasia in the keratinizing epithelium of the epidermis. Marapsin has been shown to be strongly up-regulated in the hyperplastic and hyperproliferative epidermis in psoriasis.^33^ More recently, Adachi et al.^34^ have identified marapsin in the rat cornea, which agrees with an earlier study by Wong et al.^35^ showing strong marapsin mRNA expression in the mouse eye. In normal conditions, the epidermal cell cycle is completed in about four weeks. But in psoriasis, the epidermal cell cycle is accelerated. Cell division in the basal layer occurs every 1.5 days, and the migration of keratinocytes to the stratum corneum occurs within approximately 4 days. This results in the hyperproliferation of keratinocytes.^36^ In addition, Aragona et al.^37^ showed that squamous metaplasia was present in ocular surface impression cytology specimen in patients with psoriasis without eye involvement.

The present study found thickening of the corneal epithelium without eye involvement to be associated with psoriasis. Although ocular surface involvement is absent, the authors think that increased marapsin expression and squamous metaplasia in psoriasis patients’ eyes may cause corneal epithelium hyperproliferation and thickening due to increased turnover rate.

The limitations of the present study are as follows: it is a cross-sectional study; it includes a relatively small number of participants; and it is unable to evaluate patients due to diagnosis time.

## Conclusion

The present study measured corneal epithelial thickness using contactless SD-OCT and demonstrated a positive correlation between the severity of psoriasis and CCET. The authors think that CCET may be one of the indicators of disease severity in psoriasis patients, and believe the present findings will be supported by prospective studies that include a high number of participants in the future.

## Financial support

The authors declare that no funds, grants, or other support were received during the preparation of this manuscript.

## Authors’ contributions

İrfan Botan Güneş: Material preparation, data collection and analysis; first draft of the manuscript; comments on previous versions of the manuscript; final manuscript approval.

Hakan Öztürk: Material preparation, data collection and analysis; comments on previous versions of the manuscript; final manuscript approval.

Fuat Yavrum: Material preparation, data collection and analysis; comments on previous versions of the manuscript; final manuscript approval.

Bediz Özen: Material preparation, data collection and analysis; comments on previous versions of the manuscript; final manuscript approval.

Bern Aksoy: Material preparation, data collection and analysis; comments on previous versions of the manuscript; final manuscript approval.

## Conflicts of interest

None declared.
